# Editorial Notes

**Published:** 1904-12

**Authors:** 


### Editorial Notes.

---

**University College, Bristol.**

The presence of the distinguished President of the Royal College of Physicians of London (Sir William Selby Church, Bart., K.C.B., M.D.) gave much *éclat* to the annual distribution of prizes to the students of the Faculty of Medicine on October 28th, 1904, under the chairmanship of the Right Honourable Lewis Fry. The Dean of the Faculty reported numerous distinctions obtained by Bristol students during the year, and mentioned that the Long Fox Memorial was one of the medical events of the year.

**Long Fox Memorial Lectures.**

This memorial was established by private contributions in memory of the late Dr. Edward Long Fox, and the amount contributed has been devoted partially to the endowment of a lectureship in the College, the lecture to be given annually by some person to be selected from time to time by the Trustees. The first Long Fox Lecture was given by Dr. John Beddoe, F.R.S., on November 4th, 1904, and we are gratified that we are able to give our contributors an opportunity of reading it at leisure.

**Long Fox Memorial.**

The amount subscribed by the friends of Dr. Long Fox after defraying the expenses and providing a memorial brass placed in the Bristol Cathedral, was vested in trustees for the purpose of providing (*a*) an honorarium to the lecturer, who shall be appointed annually to deliver one lecture at the University College, Bristol, on some subject connected with medicine or the allied sciences. The lecturer shall be selected from Bristol or the neighbourhood, or shall have been formerly a student or member of the teaching staff of the College. (*b*) An endowment (subject to the payment of the said honorarium to the lecturer) of the Long Fox Medical Missionary Fund, for the benefit during such time as the Appointing Board may from time to time determine while he continues at the College of a "Long Fox Medical Missionary Student," such student having been entered for not less than one academic year and registered as a medical student by the General Medical Council.

**Medical Education.** THE necessity for the maintenance of the standard in the classical education of the medical student has been happily illustrated by the Professor of Greek and Latin of the University College, Bristol, Professor Brooks, M.A., in the opening lecture of the present session, on "The Claims of the Classics," when he remarked that "it had been his privilege ever since it was first started to take in the *Stethoscope*, the organ of the Bristol Medical School. Nothing was more encouraging to a student of the classics than to observe how, in the technical articles of that journal, the vocabulary was almost wholly taken from the Greek. The small amount that was not Greek was Latin, and sometimes the two languages were combined with lawless dexterity in one and the same word. The dependence upon them was so absolute, that one was driven irresistibly to the conclusion that if the Greek and Latin languages did not fortunately already exist it would have been necessary for the purpose of medical science to invent them." When we hear protests from the unlearned as to their difficulties in comprehending the names of plants and flowers, it would appear that the fault lies not in the names of the plants, but in the want of education in the classics: it therefore becomes a duty of the scientific physician, chemist, botanist, &c., in these days when the future of the classics is in the balance, to do all in their power to maintain untarnished the inheritance of the past, and especially so as we learn, again from Professor Brooks, that "the cause of the classics is destined to survive. If it is eclipsed for a time it will have a new renascence, for its anchorage is in the general heart of mankind; it is interwoven with the stuff and fabric of human life, with the past history of man and his future destinies, his emotion and morality, his feeling for conduct, and his sense of beauty. So long as the human spirit seeks above all for human sustenance—in other words, so long as humanity remains in man—so long, I imagine, will these studies retain an eternal fascination and an eternal appeal."

Some remarks of Dr. Pye-Smith recently tend in the same direction: "Technical education is an admirable thing, but whenever a boy at school is put on the modern side because he is intended to be a merchant or a druggist, his education for its own sake, his liberal education, has ended. He may go on if well grounded to an indefinite degree of excellence, but he has already begun the important task of providing for himself and those dependent on him. His science is *Butterbrod-Wissenschaft*, and he feeds no more on ambrosia."

**The Treatment of Insanity.**

Dr. Eager, in his address to the Bristol Medico-Chirurgical Society (page 289), has recently given us a sketch of what a properly-equipped hospital for the insane might be like in the future, and a suggestion was made that such cumbrous methods as certificates might be done away with.

The difficulties in carrying out a sufficient treatment of insanity have been well described by Dr. T. Claye Shaw, in his book entitled *Ex-Cathedrâ Essays on Insanity* (Adlard & Son, 1904, p. 228), when he remarks that "the well-to-do private patient is the one who has the best chance of being successfully treated: he can have the advice of the general physician as well as that of the specialist, of the electrician, and of the chemist to examine the secretions, of the bacteriologist, and of the surgeon; for it is very certain that knowledge in those departments is essential to the true diagnosis of most forms of insanity, and therefore to the treatment. As a rule, partly owing to the expense, partly to the prejudices of the friends, it is not possible to obtain all the conditions necessary for recovery." Of late years the public have been so steeped in prejudice against all private asylums that we welcome the above views as being sound and in the best interests of the patients mentally afflicted.

**Winsley Sanatorium.**

We are authorised to inform our readers that the Sanatorium has been nearly completed. The building itself is finished and the main block in full work, and the second block will be ready for patients in a fortnight. Thus eighteen beds were thrown open on December 5th, and the remaining forty-two will be available by the second week in January next. Of the eighteen now being occupied, twelve belong to the maintained beds, and are as follows: Bristol Corporation, six; and one each to Bath Corporation, Swindon Town, G.W.R. Works, Swindon, Bradford and Trowbridge, Highworth Rural District Council, and the Clarke bed. The first two of these are proportioned to their full number to be maintained, the others have been allocated by ballot. The six non-maintained beds are open to any subscriber's nomination. For these application has to be made to the Secretary, Mr. L. A. Goss, 84 Park Street, Bristol, from whom nomination papers and certificates may be obtained, and to whom they should be returned when filled up and signed. When a bed is available the Secretary will send an intimation to the applicant if the case is suitable. For these beds a weekly sum not exceeding ten shillings a week will be charged, and the amount must he paid monthly in advance. The Secretary will give any information desired in reference to maintained beds, and to whom applications should be made for these beds.

In all cases the applicant, after being recommended by a medical practitioner, will have his reports brought before a member of the Medical Board, who will certify as to the fitness or otherwise of such applicant for treatment in the Sanatorium.

